# Supplementary material for: Quality Improvement Amid a Global Pandemic: A Virtual Curriculum for Medical Students in the Time of COVID-19
Source: MedEdPORTAL. 2021 Feb 5;17:11090. doi: 10.15766/mep_2374-8265.11090 (PMC7880258; doi:10.15766/mep_2374-8265.11090)
Supplement: Supplementary file 1 — Assessment Rubrics.docxPreceptor Facilitation Instructions.docxCurricular Outline.docxPostcourse Survey Questions.docxCourse Syllabus.docxLecture Introduction to Quality Improvement.pptxLecture Introduction to Incident Command Systems.pptxStakeholder Analysis Template.xlsxProject Charter.pptxPlan-Do-Study-Act Worksheet.pdfPeer Feedback Rubric.docxPeer Review of Presentations.docx [file _Educational_Summary_Report_11090.pdf › _Educational_Summary_Report_11090.pdf]

MedEdPORTAL® The AAMC Journal of Teaching and Learning Resources

Original Publication Open Access

Quality Improvement Amid a Global Pandemic: A Virtual Curriculum for Medical Students in the Time of COVID-19

Katelyn E. Donohue, MD*, Dara L. Farber, MD, Nidhi Goel, MD, MHS, Christopher R. Parrino, Norman F. Retener, MD, Syedmehdi Rizvi, MD, MS, Philip C. Dittmar, MD

*Corresponding author: Kdonohue@som.umaryland.edu

Abstract

Introduction: The COVID-19 pandemic in March of 2020 necessitated the removal of medical students from direct patient care activities to prevent disease spread and to conserve personal protective equipment. In order for medical student education to continue, virtual and online electives were designed and implemented expeditiously. We created a virtual curriculum that taught quality improvement (QI) skills within the context of the global pandemic. **Methods:** This 4-week curriculum enrolled 16 students. Students completed the revised QI knowledge application tool (QIKAT-R) before and after the course to assess QI knowledge. Students completed prereading, online modules, and received lectures on QI and incident command systems. Each group designed their own QI project related to our hospital system's response to the pandemic. Finally, groups presented their projects at a peer symposium and completed peer evaluations. **Results:** Students' QIKAT-R scores improved throughout the course from a mean of 5.5 ($SD = 1.3$) to a mean of 7.5 ($SD = 1.1$; $p < 0.001$). Students reported that the virtual learning experience delivered the material effectively, and all students agreed that they would participate in QI work in the future. **Discussion:** Patient safety and QI topics are content areas for multiple medical licensing examinations. Virtual learning is an effective way to deliver QI content to medical students and residents, especially when projects are trainee-led, QI-trained faculty serve as mentors, and the projects harmonize with institutional goals. Our virtual pandemic-focused curriculum has demonstrated efficacy in increasing medical student QI knowledge.

Keywords
Virtual Learning, Quality Improvement, Incident Command, Population Health, Patient Safety

Educational Objectives

By the end of this elective learners will be able to:

1. Apply new quality improvement (QI) and incident command knowledge to a pandemic-related problem.
2. Identify a small but impactful problem in the hospital or medical school related to the current public health crisis amenable to QI research.
3. Develop a process map and stakeholder analysis.
4. Write a thorough literature review and use evidence to support an intervention or innovation.
5. Apply knowledge gained in the course to develop a written project plan for a plan-do-study-act cycle that could be implemented and presented as part of the final project plan.
6. Engage with teammates to produce a final project plan and virtual platform presentation of the QI proposal.

Citation:
Donohue KE, Farber DL, Goel N, Parrino CR, Retener NF, Rizvi S, Dittmar PC. Quality improvement amid a global pandemic: a virtual curriculum for medical students in the time of COVID-19. *MedEdPORTAL*. 2021;17:11090.
https://doi.org/10.15766/mep_2374-8265.11090

Introduction

In March of 2020, the global pandemic due to the SARS-CoV-2 virus had a sudden and dramatic impact on medical education. Medical students across the United States were removed from direct patient care activities in order to limit the spread of SARS-CoV-2 and conserve personal protective equipment (PPE) based on recommendations from the AAMC.[1] Medical schools faced unprecedented pressure to create novel virtual educational experiences for students so their education could progress in the midst of the pandemic. Guidance from the AAMC as of April 14, 2020 stated:

Copyright © 2021 Donohue et al. This is an open-access publication distributed under the terms of the Creative Commons Attribution license.

> Given the magnitude of the COVID-19 pandemic's disruption to all aspects of life, including medical education, changes to the order of clinical experiences, a shortened duration of direct patient contact weeks, the expansion of alternative, nondirect patient contact clinical activities, and altered progression through the required clinical curriculum likely will occur for many students.[1]

In response to the global crisis, we expeditiously developed a virtual quality improvement (QI) curriculum for medical students that enabled them to remain involved in the efforts to combat SARS-CoV-2 despite being removed from direct patient care activities.

The ACGME requires that all residents participate in patient safety and QI (PSQI) programs as part of their training,[2] but these educational experiences are rarely longitudinal, with program directors citing time, funding, and lack of QI expertise as the major barriers to more effective implementation.[3] Integration of formal PSQI initiatives has been increasingly recognized as an vital component of medical education, and thus it is important to provide students and trainees with the fundamental knowledge and tools to understand and participate in PSQI activities.[4] Such involvement is paramount to building careers and lifelong participation in QI, and such QI involvement is part of physician maintenance of certification.[4] Early exposure to PSQI activities not only provides a foundation in requisite knowledge, but also allows for early participation in activities that can have a meaningful impact on front-line patient care.[2,4]

This QI curriculum was uniquely designed for implementation in a fully virtual format and provided students an opportunity to participate in the inner workings of the hospital's incident command system response to the pandemic. The curriculum was designed to be a 4-week fully online elective experience that medical students could use toward their graduation requirements. There is a substantial body of QI experiences that have been previously published in *MedEdPORTAL*, primarily experiential curricula to be used in residency training. There are fewer QI curricula targeting medical student learners. The only published virtual experience with QI for medical students was a patient safety simulation exercise, although this was implemented in in-person small groups.[5] Bartlett and Huerta developed a QI curriculum for medical students and demonstrated its efficacy in promoting QI knowledge, and included completion of a plan-do-study-act (PDSA) cycle.[6] There are more published QI curricula for resident learners, but most include at least one aspect of the curriculum to be completed in person.[4,7] Stewart et al. developed virtual QI modules for pediatrics residents in which residents demonstrated knowledge improvement, but the curriculum did not include a project component.[2] Despite the existence of numerous QI curricula, there is a paucity of literature combining QI with a response to global, national, or local catastrophes. Phillips et al. proposed that the fusion of QI and emergency preparedness is essential to providing quality and safe care for patients in the resource-constrained environment which often accompanies disasters, both natural and human-made.[8] There were only three prior *MedEdPORTAL* educational modules that incorporated pandemic medicine for medical students.[9-11] We envisioned our curriculum as an important step in bridging the gap in the literature and in clinical practice between the application of QI in the setting of a global pandemic where virtual education is quickly finding its niche in the medical student curriculum.

Numerous online resources are being developed for educators and students to support continued learning in these unprecedented times.[12] Initial recommendations from the AAMC in 2007 on the use of educational technologies proposed that computer-aided instruction and virtual patients may be effective for facilitating basic knowledge acquisition, improving decision making, and enhancing perceptual variation.[13] Virtual learning scenarios and virtual patients were subsequently shown to promote the application of foundational knowledge and development of clinical reasoning strategies.[14] In addition, these authors suggested that matching cognitive demand in virtual patient exercises with medical student capabilities may improve medical student mental health through improving intrinsic motivation, thereby positively impacting the overall learning experience.[14] The virtual learning environment has been shown to improve student satisfaction, engagement, and recall of key educational topics;[15] intrinsic motivation as well as active learning strategies of medical student learners are predictive of academic success.[16] Virtual classrooms have been employed successfully in the past for high-fidelity infection control training in the setting of the 2014 Ebola epidemic.[17]

Our pandemic-focused QI elective combined QI with exposure to incident command systems in the midst of a global pandemic, a subject not extensively covered in medical school curricula[18] in a feasible and easily implementable curriculum. In addition, this course was implemented entirely via a virtual platform during a time that necessitated remote learning to maintain safety and conserve PPE. Our course allowed medical students to develop impactful projects relevant to the current public health crisis while uniquely combining QI and exposure to the complex response of

Copyright © 2021 Donohue et al. This is an open-access publication distributed under the terms of the Creative Commons Attribution license.

our hospital's incident command team. A possible explanation for the paucity of this combination in the medical education literature is that disasters are often thought of as limited in time, such that the incident has resolved by the time that a QI initiative can be designed, implemented, and its impact measured. QI and incident command systems are often functionally separate entities within hospital systems with different leadership, which contributes to possible limits in communication and coordination at the outset of a disaster.[8] The current COVID-19 pandemic is unlikely to abate quickly, offering an ideal opportunity for students to participate in the hospitals response in conjunction with QI.

Methods

Curricular Context

This educational experience was created and implemented in response to the global COVID-19 pandemic and the urgent removal of all medical students from direct patient care. It was designed in March of 2020 as a 4-week elective that would fulfill the Liaison Committee on Medical Education's requirements for an elective rotation for medical students and was approved by our curriculum committee. Learners included both third- and fourth-year medical students. Prerequisites for enrollment in the experience were the same as for our electives with direct patient care: students were required to have completed the preclinical curriculum and have passed Step 1 of the USMLE. There were no specific prerequisite QI experiences or knowledge required.

Curricular Development

The circumstances surrounding the COVID-19 pandemic necessitated that this curriculum be developed more rapidly than most others. Existing QI experiences offered to medical students and residents at our institution were examined for adaptability to a virtual experience. Resources that could be viewed virtually, including lectures given by faculty members, were assembled to provide an appropriate knowledge base for conducting QI projects. Experiences that were typically done in person were broken down into components, and any aspects of the existing curriculum that could be completed virtually were included. The experience of implementing a QI project was mimicked by having the students develop all the individual components of the QI project, including background research, intervention, and outcome measures, and ultimately presenting their work at a symposium with their peers. Additionally, novel curriculum elements were designed to provide a specific focus on the institution's response to the global pandemic, with particular attention paid to logistical constraints and need for rapid decision-making. The assessment measures for each component of the curriculum were designed by two faculty with extensive experience in QI and educational assessment measures, and were approved by all participating faculty members. Each item in the assessment rubric (Appendix A) was designed to map directly to course objectives. As there were multiple faculty members serving as facilitators for the course, the rubrics were designed to be clear and simple for every facilitator. The entire curriculum, including assessment measures, was evaluated by experts in QI and approved by the institutional curriculum committee.

Implementation

An email detailing the virtual elective was sent to all third- and fourth-year medical students, and students were enrolled in the course in the order in which they expressed interest, to a maximum capacity of 16 students. The entirety of the course was conducted via Microsoft Teams, an online platform used for group meetings by the University of Maryland School of Medicine. All lectures and group meetings were conducted virtually. Students submitted all assignments via the online platform, including their final presentations. All faculty involvement was voluntary. Course leaders identified ideal faculty members to serve as group facilitators 2 weeks prior to the start of the course. Faculty members were initially recruited from the department of medicine via email, and faculty were informed that the time commitment would be 3-4 hours per week for a total of 4 weeks. Faculty members with a background in QI, patient safety, or medical education were selected as group facilitators. Course leaders created a detailed course facilitation guide for faculty members (Appendix B) and sent weekly reminder emails to faculty to remind them of their upcoming obligations to the course. The curriculum was designed to be implemented in four 1-week blocks, with each week encompassing a different aspect of QI work (Appendix C). Students were assigned to teams of four students, and each team was paired with a faculty facilitator. Teams were assigned by the course leaders with the goal of having diversity in gender, race, and scholastic performance. Google Forms, a free online software program, was used to create the final postcourse survey (Appendix D). The survey was administered to students via email, and responses were anonymous.

Session Details

At the beginning of week 1, students completed the preintervention revised QI knowledge application tool (QIKAT-R).[19] Course facilitators met with students virtually; they reviewed the syllabus (Appendix E), course objectives, and expectations for all future assignments and answered any learner questions. Students were required to read a journal article by Silver et al.[20] on initiating a QI project and recommended to

Copyright © 2021 Donohue et al. This is an open-access publication distributed under the terms of the Creative Commons Attribution license.

complete the Institute for Healthcare Improvement's modules #QI 101-105 and #Patient Safety 101-104,[21] which are publicly available and freely accessible online. They were also provided in the syllabus with several additional resources, such as podcasts, to complete during the duration of the course (Appendix E). Students attended the institution's COVID-19 incident command briefing and patient safety huddle daily, as well as the weekly hospital-wide COVID-19 briefing, all of which were conducted virtually. Each student group was expected to meet virtually with their group facilitator at the beginning of each week for guidance on deliverables, and at the conclusion of the week for feedback on the finished deliverables. There was not a prescribed amount of time for meetings with the group facilitators, but they typically lasted 45-60 minutes.

In the first week, students participated in an introduction to QI lecture (Appendix F), as well as an introduction to incident command lecture (Appendix G). Using the information from the background readings, lectures, and daily incident command briefings, each group identified a QI problem related to the COVID-19 pandemic response at the institution. Groups met virtually with their faculty facilitators to ensure the problem was appropriate and subsequently submitted a problem identification synopsis, stakeholder analysis, and project charter (Appendices H and I). Each group facilitator reviewed the project charter and problem synopsis and provided written and verbal feedback to their group at the conclusion of week 1.

During week 2, student groups completed a literature review of the problem they chose to address and developed an evidence-based intervention. Students were expected to use QI principles, the daily incident command briefings they attended, and as much evidence as possible to support their choice of intervention. Learners then completed a process map analyzing factors contributing to their chosen problem. Groups met with their facilitators virtually and received written feedback on their literature review and process map, based on the included rubric (Appendix A), prior to moving on to week 3.

Throughout week 3, each group designed a PDSA cycle that could be implemented to test their problem and proposed intervention. Although full implementation of a traditional PDSA cycle while physically removed from the hospital was not feasible, each group completed a PDSA worksheet (Appendix J) and authored and submitted a full report of how they would implement the PDSA cycle. Included in this submission were identification of roles, details of study design, strategies to track outcomes, and a proposal of how to expand the scope of the intervention if success was noted.

During the final week of the elective, each team prepared a formal write-up of their project and created a platform presentation for their peers. These presentations were given on the final day of the course in a virtual symposium. Groups uploaded their final presentations to the course website and completed peer evaluations of the students on their team (Appendix K) as well as evaluations of presentations by other groups (Appendix L). Final grades for the course consisted of scores received on the project submissions by both faculty and other students, as well as the peer evaluations completed within each group. Comments from peer evaluations were anonymized and included as part of the final evaluation.

Outcomes Evaluation

To evaluate student understanding of QI concepts, we administered the QIKAT-R[19] prior to, and at the conclusion of the elective. The QIKAT-R is a validated tool to assess learners QI knowledge, and ability to apply that knowledge to a complex problem and propose a solution.[19] It is a case-based assessment tool that has been used in both resident and student assessment of QI curricula, specifically to assess achievement of objectives related to QI knowledge application. The QIKAT-R's assessment of QI knowledge application maps to learning objectives 1 through 5, which required learners to apply QI knowledge learned from lectures and readings. For each of the two assessments, students submitted typed responses via Microsoft Teams to three assigned QIKAT-R cases. Responses were blinded and graded by course directors. Each student was assigned a unique identifier so responses could be anonymously matched for pre- and postcourse comparison. Students' QIKAT-R scores were not incorporated into their final assessment and grading.

To assess the overall effectiveness of the course, students were asked to complete a voluntary and anonymous course evaluation (Appendix D) using a 5-point Likert scale survey. The survey questions assessed their use of the readings, modules, and lectures, their opinions on the helpfulness and relevance of these tools, their perceptions of change of their QI knowledge, and any likelihood of engaging in future QI projects. The survey concluded with an optional free-text response section for comments.

In accordance with the institution's grading policy during the COVID-19 pandemic, the students were graded on a pass/fail basis. Their grade consisted of rubric evaluations of weekly assignments (Appendix A), peer evaluations of their final projects (Appendix L), as well as peer evaluations of their participation in group activities (Appendix K).

Copyright © 2021 Donohue et al. This is an open-access publication distributed under the terms of the Creative Commons Attribution license.

Results

A total of 16 students enrolled in the course, with four students in each group. All students completed all elements of the curriculum and passed the course. Student QI projects included optimizing response times to troubleshoot broken video communication devices on units with patients with COVID-19; ensuring that return-to-work guidelines for hospital employees were clear, consistent, and readily available; conserving PPE by using checklists and spotters; and limiting workplace exposure to COVID-19 by screening employees daily for fever upon entrance to the hospital.

A paired sample *t* test was conducted to compare QIKAT-R scores before and after participating in the course as a marker of QI knowledge. There was a significant difference observed in the scores, with a mean precourse score of 5.5 ($SD = 1.3$) and mean postcourse score of 7.5 ($SD = 1.1$); $t\,(15) = 2.13$, $p < .001$.

All 16 students completed the optional postcourse survey. Quantitative feedback from the survey indicated that the course was the first QI experience for all students (Table). Of students, 100% agreed that their knowledge of QI improved substantially as a result of the elective, answering *strongly agree* (5 of 5) or *agree* (4 of 5). All students found the virtual learning experience to be an effective way to deliver course content and felt more connected to hospital-wide efforts to combat COVID-19. Finally, all students answered that they would be more likely to engage in future QI work after taking this course. The overall average response across all questions on the 5-point Likert scale was a 4.8 out of 5.

Table. Average Postcourse Survey Responses ($N = 16$)

Question	*M* Rating[a]
The Institute for Healthcare Improvement modules and readings were valuable in improving my knowledge of quality improvement.	4.6
I found the daily hospital wide safety huddle to be a valuable learning experience.	4.8
The introduction to QI lecture improved my QI knowledge.	4.9
The introduction to disaster medicine lecture improved my knowledge of disaster medicine.	4.5
My knowledge of QI has improved substantially as a result of participating in this elective.	4.9
I found the virtual learning experience, including the software used, to be an effective way to deliver the course content.	4.8
I felt more connected to the hospital-wide efforts to combat the COVID-19 pandemic by participating in this elective.	4.9
I would recommend this elective to my colleagues as a valuable learning experience.	4.8
As a result of participating in this elective, I am more likely to engage in QI work in the future.	4.6

Abbreviation: QI, quality improvement.
[a]Rated on a 5-point Likert scale (1 = *strongly disagree*, 5 = *strongly agree*).

Qualitative feedback from learners indicated that this elective was well received. The learners appreciated the opportunity to participate in the hospital's pandemic response and reported increased connectedness with the hospital response despite being physically distant. Students reported that the expectations were clear, and that the course was well organized and implemented despite being entirely virtual. Learners reported that the virtual format was effective, but some did report wishing there were additional virtual meetings with faculty.

When asked what the most positive and negative aspects of the curriculum were, student comments were centered around three main themes: connectedness to the hospital response, virtual platform for learning, and increase in QI knowledge.

Connectedness to the hospital response: Students reported feeling connected to their peers and the greater response of the hospital to the COVID-19 pandemic. This was important not only for a meaningful educational experience, but also personal well-being during an isolating and challenging time. Sample comments included:

- "I found that this elective was a great lifeline into the hospital and really bolstered my understanding of how things get done, administratively, at University of Maryland Medical Center."
- "I absolutely loved this elective. The online modules were perfect and I really liked feeling more connected to the COVID-19 crisis management plans. I wish there were more meetings in which we could all gather together to discuss as a big group."

Virtual platform for learning: Learners felt as if the virtual format of this curriculum was structured in a way to provide good education related to QI and pandemic medicine. Some students wanted more live interactions with faculty members. Sample comments included:

- "This elective was super conducive to doing from home; I didn't feel like my experience was incomplete due to lack of in person meetings/lectures."
- "I would have appreciated more live lectures and/or interactive discussion sessions."

Increase in QI knowledge: Students thought their QI knowledge was enhanced as a result of this course through a combination of live lectures, online modules, and working through the project itself. Sample comments included:

Copyright © 2021 Donohue et al. This is an open-access publication distributed under the terms of the Creative Commons Attribution license.

- "I really enjoyed working on a project related to the current pandemic and learning more about various efforts in the hospital that focus on improving patient care and safety during this challenging time."
- "...The QI component of the course was also insightful and helped me develop my understanding of how projects are implemented and changes are made."

Discussion

The COVID-19 pandemic has dramatically reshaped the landscape of medical education in record time, leaving medical schools forced to scramble to create novel educational experiences for their students. Our hope is that this virtualized QI curriculum can help fill this need at other institutions, and ultimately be a resource for a fully virtual QI curriculum in any context. Both medical students and faculty have a vested interest in ensuring adequate exposure to QI, as content for the USMLE exams includes patient safety and QI.[22] Many other QI curricula have been developed for trainees with success and positive feedback both from trainees and faculty.[6,7,23-24] QI competency can be achieved through implementation of an established and systematic curriculum.[7] Prior literature has identified three factors that are critical to establishing a successful curriculum: projects with opportunities for trainees to present and publish, QI-focused or formally credentialed mentors, and projects aligned with institutional goals.[7] This course met these benchmarks by giving learners autonomy in project determination and the opportunity to participate in a peer symposium. The inclusion of QI-experienced faculty has provided students with ample resources and guidance to have a meaningful experience. The creation of this course at a time when the COVID-19 pandemic has placed a strain on hospital resources and required the timely development of new safety measures to optimize delivery of health care and keep our health care providers safe provided students a unique opportunity to work with hospital leadership.

Our curriculum was able to increase learner knowledge and proficiency with QI projects as evidenced by increased scores on the QIKAT-R. This improvement in scores has been observed in prior, similar QI courses. Bartlett and Huerta[6] developed a longitudinal 4-year PSQI curriculum for medical students and demonstrated a 2.1-point increase in the QIKAT-R score across their cohort as a result of their course. Reardon et al.[23] created a similar curriculum for psychiatry residents and observed statistically significant improvement of 3.3 points in the QIKAT-R scores of their residents. Medical students in our course demonstrated an improvement of 2.0 points on the QIKAT-R after 4 weeks, suggesting the efficacy of this course in increasing learner knowledge of QI topics that is equivalent to other QI curricula, including those that are not fully virtual.

The course was well received by students and rated highly in the postcourse survey. Students agreed that virtual learning was both possible and effective to communicate course information and achieve course objectives. In a time when physical distancing is a priority and in-person meetings are less likely, it has proven feasible to create a course that educates students in QI topics and connects these students with our hospital system's coordinated response to the pandemic. Several of our students during the first iteration of the course were involved with our Medical Student Supplementary Labor Force in various volunteer efforts remotely or in the hospital without direct patient contact. These students used their experiences with the various pandemic relief efforts as inspiration for their QI projects. Enabling medical students to remain engaged with the medical community and their hospital system's response to the COVID-19 pandemic fostered a sense of purpose for the learners, which is highly valued in a time of great uncertainty.

While we believe this course was a valuable addition to the current repertoire of virtual electives in the education literature, the analysis of the course effectiveness was limited by our pilot sample of 16 students. The improvement in QIKAT-R score was substantial and similar to other QI courses, but would benefit from data collected on subsequent administrations of this curriculum. An additional limitation was the lack of face-to-face faculty contact with students. While the relatively minimal time requirement by faculty preceptors was ideal in the setting of increased clinical demand, it must be balanced with providing students adequate facilitation support. Students rated the course highly, but several students proposed additional virtual meetings with faculty as a potential future improvement to the course. Some of the comments may be attributed to the virtual format, but in future iterations of this course, we will encourage faculty to have more frequent meetings with students to increase the number of student-faculty interactions.

The authors faced a number of challenges as this course was implemented. We hope by sharing these challenges, this curriculum will be easy to utilize by other institutions. Our first challenge was the rapid turnaround time from conception to implementation. Students were removed from clinical rotations with little notice, and the need to replace their education with virtual alternatives was immediate. Given the limited time for planning, we relied on elements of current, mainly QI, electives at our institution, and worked to quickly convert existing elements into a fully virtual curriculum. Additionally, we used concomitant

Copyright © 2021 Donohue et al. This is an open-access publication distributed under the terms of the Creative Commons Attribution license.

institutional changes to our advantage, and integrated students into our hospital's incident command response. We would encourage educators to brainstorm creative solutions to integrate students into the larger hospital response to a crisis, rather than attempt to create curricula that pulls them away from it.

Similarly, a major challenge facing our education team was engagement of students in a virtual curriculum while they were experiencing isolation and facing significant stressors. We addressed this challenge in two ways, first by having the students work in groups, and second by integrating them into the hospital response. Team projects required students to engage with their peers; this was reinforced by grading that included peer evaluation as a portion of their final grade. By including our students in the incident command response, they were able to feel connected to the hospital and medical school, which we felt was extremely important for both a meaningful educational experience and also their well-being. We also encouraged students previously involved in volunteer projects related to the hospital COVID-19 response to integrate their volunteer efforts with their QI projects, to further emphasize a sense of purpose, and to make the curriculum more meaningful to them. As mentioned previously, students proposed increased meetings with faculty as a potential area for improvement in the course. This reflected the challenge of engaging faculty at a time when most faculty members' clinical duties were maximized, or they were facing the challenging task of being redeployed to meet a patient surge. This was arguably the biggest challenge given the finite number of faculty members available. Recruitment efforts were initially focused on faculty members who were extensively involved in resident and student education, and felt a sense of commitment to their continued education despite a global crisis. The facilitator role was also designed to be as straightforward as possible by providing clear instructions and simple rubrics, so leading the student team and providing feedback was attainable within their busy schedules. Despite the limitations in time and resources, faculty facilitators were engaged and added meaningfully to the students' experience within the course.

A potential limitation to implementing this course was involvement of faculty members who were experienced in medical education and QI. Some institutions may not have access to a large number of faculty with QI experience, which would make providing feedback to students challenging. The authors suggest that in this instance, faculty members without equivalent QI experience could take the Institute for Healthcare Improvement modules,[21] or a similar structured QI course, to give them the necessary background experience. A structured QI course, in combination with the background readings, should provide faculty members with the necessary expertise to become facilitators. Ideally facilitators with less QI knowledge and experience could be paired with more experienced faculty so they could gain experience over several iterations of the curriculum.

Future directions for this syllabus include implementation of this material as part of the first- and second-year medical school curriculum, especially if the pandemic necessitates virtual lectures and limited in-person sessions for these students. Revision of the curriculum in accordance with student feedback will also be completed after each iteration. Our hope is that this curriculum could be replicated at other medical schools across the country, as the pandemic has quickly transformed medical student learning nationwide to rely more heavily on virtual education. As the COVID-19 pandemic progresses, it is likely that medical students' direct patient care activities will remain limited in some capacity, requiring medical schools to provide a repertoire of virtual educational experiences. This curriculum also has the potential to expand beyond medical school and have an impact on graduate medical education in residency. Finally, we envision this curriculum as being applicable for any human-made or natural disaster that may occur worldwide or at any institution and that this curriculum may be tailored toward such events. Additionally, this curriculum could be adapted and implemented in many environments, including any situations that necessitate distance learning, such as parental leave, student illness, or situations in which a virtual QI curriculum could be used to complement a clinical experience.

Appendices

A. Assessment Rubrics.docx
B. Preceptor Facilitation Instructions.docx
C. Curricular Outline.docx
D. Postcourse Survey Questions.docx
E. Course Syllabus.docx
F. Lecture Introduction to Quality Improvement.pptx
G. Lecture Introduction to Incident Command Systems.pptx
H. Stakeholder Analysis Template.xlsx
I. Project Charter.pptx
J. Plan-Do-Study-Act Worksheet.pdf
K. Peer Feedback Rubric.docx
L. Peer Review of Presentations.docx

All appendices are peer reviewed as integral parts of the Original Publication.

Copyright © 2021 Donohue et al. This is an open-access publication distributed under the terms of the Creative Commons Attribution license.

Katelyn E. Donohue, MD: Assistant Professor, Departments of Medicine and Pediatrics, University of Maryland School of Medicine; ORCID: https://orcid.org/0000-0002-1168-6074

Dara L. Farber, MD: Assistant Professor, Departments of Medicine and Pediatrics, University of Maryland School of Medicine

Nidhi Goel, MD, MHS: Assistant Professor, Departments of Medicine and Pediatrics, University of Maryland School of Medicine

Christopher R. Parrino: Fourth-year Medical Student, University of Maryland School of Medicine

Norman F. Retener, MD: Assistant Professor, Department of Medicine, University of Maryland School of Medicine

Syedmehdi Rizvi, MD, MS: Director of Emergency Management, University of Maryland Medical Center

Philip C. Dittmar, MD: Assistant Professor, Department of Medicine, University of Maryland School of Medicine

Disclosures
None to report.

Funding/Support
None to report.

Ethical Approval
Reported as not applicable.

References

1. Whelan A, Prescott J, Young G, Catanese V, McKinney R. *Guidance on Medical Students' Participation in Direct Patient Contact Activities. Association of American Medical Colleges*. Published 2020. Accessed May 26, 2020. https://www.aamc.org/system/files/2020-04/meded-April-14-Guidance-on-Medical-Students-Participation-in-Direct-Patient-Contact-Activities.pdf
2. Stewart D, Lye C, Lopez M, Mothner B, Camp E, Vachani J. Engaging learners through modules in quality improvement and patient safety. *MedEdPORTAL*. 2016;12:10482. https://doi.org/10.15766/mep_2374-8265.10482
3. Mann KJ, Craig MS, Moses JM. Quality improvement educational practices in pediatric residency programs: survey of pediatric program directors. *Acad Pediatr*. 2014;14(1):23-28. https://doi.org/10.1016/j.acap.2012.11.003
4. Keefer P, Orringer K, Vredeveld J, Warrier K, Burrows H. Developing a quality improvement and patient safety toolbox: the curriculum. *MedEdPORTAL*. 2016;12:10385. https://doi.org/10.15766/mep_2374-8265.10385
5. Worsham C, Swamy L, Gilad A, Abbott J. Quality improvement virtual practicum: the QI simulator. *MedEdPORTAL*. 2018;14:10670. https://doi.org/10.15766/mep_2374-8265.10670
6. Bartlett CS, Huerta SA. Creating change: an experiential quality improvement and patient safety curriculum for medical students. *MedEdPORTAL*. 2018;14:10660. https://doi.org/10.15766/mep_2374-8265.10660
7. Reed D, Wittich C, Drefahl M, McDonald F. A quality improvement curriculum for internal medicine residents. *MedEdPORTAL*. 2009;5:7733. https://doi.org/10.15766/mep_2374-8265.7733
8. Phillips S, Hughes R, Savitz LA. Synergistic opportunity to connect quality improvement and emergency preparedness. In: *Patient Safety and Quality: An Evidence-Based Handbook for Nurses.* AHRQ Publication No. 08-0043. Agency for Healthcare Research and Quality; 2008. https://www.ncbi.nlm.nih.gov/books/NBK2651/
9. Lindemann J, Owens M, Qualm C, Frentz S, Kevghas C. Pandemic scenario tabletop exercise. *MedEdPORTAL*. 2007;3:692. https://doi.org/10.15766/mep_2374-8265.692
10. Mahoney J, Suyama J, Offen B. Pandemic influenza preparedness: a patient care and team training simulation. *MedEdPORTAL*. 2009;5:7887. https://doi.org/10.15766/mep_2374-8265.7887
11. Drowos J, Lizotte-Waniewski M, Louda D. Pandemic influenza: what if? *MedEdPORTAL*. 2015;11:10162. https://doi.org/10.15766/mep_2374-8265.10162
12. *Clinical Teaching and Learning Experiences without Physical Patient Contact*. Association of American Medical Colleges. Accessed May 26, 2020. https://icollaborative.aamc.org/collection/covid19-alternative-learning-experiences/
13. *Effective Use of Educational Technology in Medical Education.* Association of American Medical Colleges; 2007. https://store.aamc.org/downloadable/download/sample/sample_id/111/
14. Berman NB, Durning SJ, Fischer MR, Huwendiek S, Triola MM. The role for virtual patients in the future of medical education. *Acad Med.* 2016;91(9):1217-1222. https://doi.org/10.1097/acm.0000000000001146
15. Ryan E, Poole C. Impact of virtual learning environment on students' satisfaction, engagement, recall, and retention. *J Med Imaging Radiat Sci.* 2019;50(3):408-415. https://doi.org/10.1016/j.jmir.2019.04.005
16. Nabizadeh S, Hajian S, Sheikhan Z, Rafiei F. Prediction of academic achievement based on learning strategies and outcome expectations among medical students. *BMC Med Educ.* 2019;19(1):99. https://doi.org/10.1186/s12909-019-1527-9
17. Sansom C. The virtual classroom and Ebola preparedness. *Lancet Infect Dis.* 2015;15(5):515-516. https://doi.org/10.1016/s1473-3099(15)70172-6
18. Pfenninger EG, Domres BD, Stahl W, Bauer A, Houser CM, Himmelseher S. Medical student medicine education: the development of an educational resource. *Int J Emerg Med.* 2010;3(1):9-20. https://doi.org/10.1007/s12245-009-0140-9
19. Singh MK, Ogrinc G, Cox KR, et al. The quality improvement knowledge application tool revised (QIKAT-R). *Acad Med.* 2014;89(10):1386-1391. https://doi.org/10.1097/acm.0000000000000456

Copyright © 2021 Donohue et al. This is an open-access publication distributed under the terms of the Creative Commons Attribution license.

20. Silver SA, Harel Z, McQuillan R, et al. How to begin a quality improvement project. *Clin J Am Soc Nephrol.* 2016;11(5):893-900. https://doi.org/10.2215/cjn.11491015
21. IHI open school online courses. Institute for Healthcare Improvement. Published 2009. Updated June 10, 2019. Accessed June 10, 2020. http://www.ihi.org/OnlineCourses
22. Federation of State Medical Boards of the United States, National Board of Medical Examiners. *USMLE Content Outline*. United States Medical Licensing Examination. Published 2020. Accessed May 31, 2020. https://www.usmle.org/pdfs/usmlecontentoutline.pdf
23. Reardon CL, Hafer R, Langheim FJP, et al. A quality improvement curriculum for psychiatry residents. *MedEdPORTAL.* 2020;16:10870. https://doi.org/10.15766/mep_2374-8265.10870
24. Manning MW, Bean EW, Miller AC, et al. Using medical student quality improvement projects to promote evidence-based care in the emergency department. *West J Emerg Med.* 2018;19(1):148-157. https://doi.org/10.5811/westjem.2017.9.35163

Received: June 24, 2020
Accepted: October 27, 2020
Published: February 5, 2021

Copyright © 2021 Donohue et al. This is an open-access publication distributed under the terms of the Creative Commons Attribution license.
