# Supplementary material for: Quality Improvement Amid a Global Pandemic: A Virtual Curriculum for Medical Students in the Time of COVID-19
Source: MedEdPORTAL. 2021 Feb 5;17:11090. doi: 10.15766/mep_2374-8265.11090 (PMC7880258; doi:10.15766/mep_2374-8265.11090)
Supplement: Supplementary file 1 — Assessment Rubrics.docxPreceptor Facilitation Instructions.docxCurricular Outline.docxPostcourse Survey Questions.docxCourse Syllabus.docxLecture Introduction to Quality Improvement.pptxLecture Introduction to Incident Command Systems.pptxStakeholder Analysis Template.xlsxProject Charter.pptxPlan-Do-Study-Act Worksheet.pdfPeer Feedback Rubric.docxPeer Review of Presentations.docx [file J. Plan-Do-Study-Act Worksheet.pdf › J. Plan-Do-Study-Act Worksheet.pdf]

QI Essentials Toolkit:

# PDSA Worksheet

The Plan-Do-Study-Act (PDSA) cycle is a useful tool for documenting a test of change. Running a PDSA cycle is another way of saying testing a change — you develop a plan to test the change (Plan), carry out the test (Do), observe, analyze, and learn from the test (Study), and determine what modifications, if any, to make for the next cycle (Act).

Fill out one PDSA worksheet for each change you test. In most improvement projects, teams will test several different changes, and each change may go through several PDSA cycles as you continue to learn. Keep a file (either electronic or hard copy) of all PDSA cycles for all the changes your team tests.

***IHI's QI Essentials Toolkit*** includes the tools and templates you need to launch and manage a successful improvement project. Each of the nine tools in the toolkit includes a short description, instructions, an example, and a blank template. NOTE: Before filling out the template, first save the file on your computer. Then open and use that version of the tool. Otherwise, your changes will not be saved.

- Cause and Effect Diagram
- Driver Diagram
- Failure Modes and Effects Analysis (FMEA)
- Flowchart
- Histogram
- Pareto Chart
- **PDSA Worksheet**
- Project Planning Form
- Run Chart & Control Chart
- Scatter Diagram

Copyright © 2017 Institute for Healthcare Improvement. All rights reserved. Individuals may photocopy these materials for educational, not-for-profit uses, provided that the contents are not altered in any way and that proper attribution is given to IHI as the source of the content. These materials may not be reproduced for commercial, for-profit use in any form or by any means, or republished under any circumstances, without the written permission of the Institute for Healthcare Improvement.

# Instructions

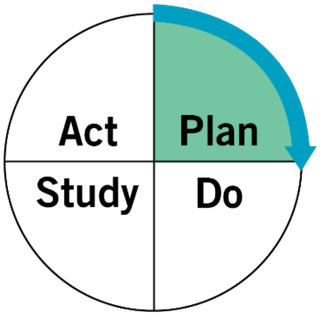


**Plan:** Plan the test, including a plan for collecting data.

- State the question you want to answer and make a prediction about what you think will happen.
- Develop a plan to test the change. (Who? What? When? Where?)
- Identify what data you will need to collect.

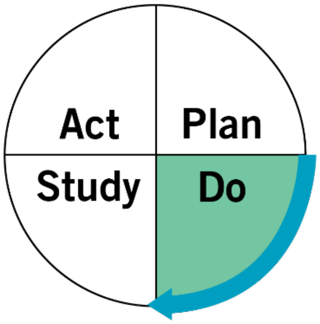


**Do:** Run the test on a small scale.

- Carry out the test.
- Document problems and unexpected observations.
- Collect and begin to analyze the data.

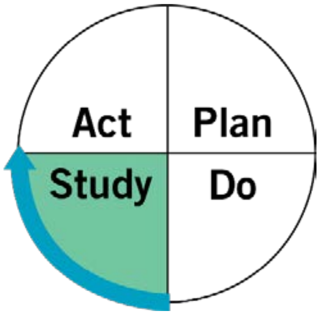


**Study:** Analyze the results and compare them to your predictions.

- Complete, as a team, if possible, your analysis of the data.
- Compare the data to your prediction.
- Summarize and reflect on what you learned.

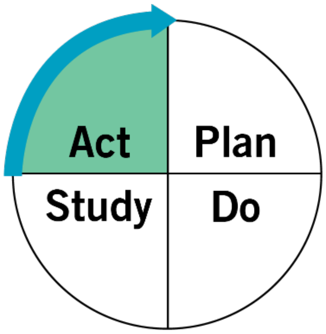


**Act:** Based on what you learned from the test, make a plan for your next step.

- Adapt (make modifications and run another test), adopt (test the change on a larger scale), or abandon (don’t do another test on this change idea).
- Prepare a plan for the next PDSA.

# Example: PDSA Worksheet

**Objective:** Test using Teach-Back (a closed-loop communication model, in which the recipient of information repeats the information back to the speaker) with a small group of patients, in hopes of improving patients' understanding of their care plans.

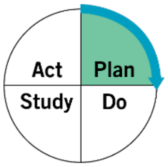


**1. Plan:** Plan the test, including a plan for collecting data.

**Questions and predictions:**

- How much more time will it take to use Teach-Back with patients? It will take more time at first (5 to 10 minutes per patient), but we will start to learn better communication skills and get more efficient.
- Will it be worthwhile? The extra time will feel worthwhile (and possibly prevent future rework).
- What will we do if the act of "teaching back" reveals a patient didn't understand the care plan? If a patient is not able to explain his or her care plan, we will need to explain it again, perhaps in a different way.

**Who, what, where, when:**

On Monday, each resident will test using Teach-Back with the last patient of the day.

**Plan for collecting data:**

Each resident will write a brief paragraph about their experience using Teach-Back with the last patient.

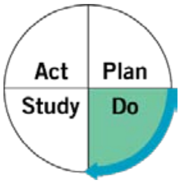


**2. Do:** Run the test on a small scale.

**Describe what happened. What data did you collect? What observations did you make?**

Three residents attempted Teach-Back at the end of the day on Monday. Two residents did not find anything they needed to ask patients to Teach-Back. Jane found that her patient did not understand the medication schedule for her child. They were able to review it again and, at the end, Jane was confident the mother was going to be able to give the medication as indicated.

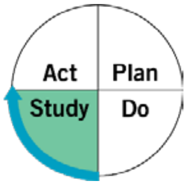


**3. Study:** Analyze the results and compare them to your predictions.

**Summarize and reflect on what you learned:**

- Prediction: It will take more time at first (5 to 10 minutes per patient), but we will start to learn better communication skills and get more efficient. *Result: Using Teach-Back took about 5 minutes per patient.*
- Prediction: The extra time will feel worthwhile (and possibly prevent future rework). *Result: Jane felt the time she invested in using Teach-Back significantly improved the care experience.*
- Prediction: If a patient is not able to explain his or her care plan, we will need to explain it again, perhaps in a different way. *Result: After a second review of the medication orders, the patient was able to Teach-Back the instructions successfully*.

In addition to the team confirming all three predictions, Jane realized the medication information sheets she had been handing out to parents weren't as clear as she thought. She realized these should be re-written — maybe with the input of some parents.

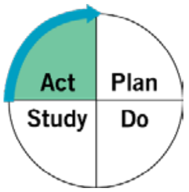


**4. Act:** Based on what you learned from the test, make a plan for your next step.

**Determine what modifications you should make — adapt, adopt, or abandon:**

Jane is planning to use Teach-Back any time she prescribes medication. Although it may take more time, she now understands the importance. The other residents are going to work on using Teach-Back specifically for medications for the next week.

They would like to pull together a team to work on some of the medication information sheets with parent input, but they are first going to gather more information through more interactions in the coming days.

Before filling out the template, first save the file on your computer. Then open and use that version of the tool. Otherwise, your changes will not be saved.

# Template: PDSA Worksheet

## Objective:

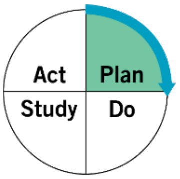


**1. Plan:** Plan the test, including a plan for collecting data.

**Questions and predictions:**

-
-

**Who, what, where, when:**

**Plan for collecting data:**

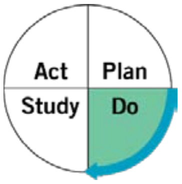


**2. Do:** Run the test on a small scale.

**Describe what happened. What data did you collect? What observations did you make?**

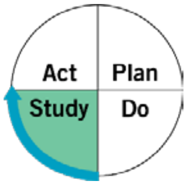


**3. Study:** Analyze the results and compare them to your predictions.

**Summarize and reflect on what you learned:**

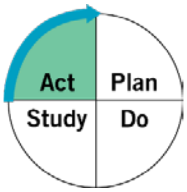


**4. Act:** Based on what you learned from the test, make a plan for your next step.

**Determine what modifications you should make — adapt, adopt, or abandon:**
