# Supplementary material for: Quality Improvement Amid a Global Pandemic: A Virtual Curriculum for Medical Students in the Time of COVID-19
Source: MedEdPORTAL. 2021 Feb 5;17:11090. doi: 10.15766/mep_2374-8265.11090 (PMC7880258; doi:10.15766/mep_2374-8265.11090)
Supplement: Supplementary file 1 — Assessment Rubrics.docxPreceptor Facilitation Instructions.docxCurricular Outline.docxPostcourse Survey Questions.docxCourse Syllabus.docxLecture Introduction to Quality Improvement.pptxLecture Introduction to Incident Command Systems.pptxStakeholder Analysis Template.xlsxProject Charter.pptxPlan-Do-Study-Act Worksheet.pdfPeer Feedback Rubric.docxPeer Review of Presentations.docx [file mep_2374-8265.11090-s001.zip › A. Assessment Rubrics.docx]

**Assessment Rubrics:** The maximum point value for each assignment is 10 points.

|  | **1pts** | **5pts** | **10pts** |
| --- | --- | --- | --- |
| **Brief Problem Identification Synopsis**  **Due: [ ]** | QI question not amenable to a small PDSA cycle. Stakeholder identification is missing. | Identified problem is workable but requires significant revision by facilitator to be amenable to a PDSA cycle, or stakeholders are incompletely identified. | Problem is amenable to a small PDSA cycle and is impactful. All stakeholders are identified. Synopsis describes how the problem is specific, measurable, and stretchable. Problem Charter is included. |
| **Literature Review & Process Map**  **Due: [ ]** | Assignment was late or missing. Literature review is grossly incomplete. | Literature review is mostly complete but missing 1 or 2 important elements, such as insufficient sources, poor research on current processes. | Written review is clear and well researched. Appropriate parties have been interviewed. Information on processes currently in place has been included. Process map is included. |
| **PDSA cycle design & PDSA worksheet**  **Due: [ ]** | Assignment was late or missing. Large areas of oversight missing in design of PDSA cycle. | PDSA cycle is mostly complete but some materials may be missing or there is insufficient detail. Roles are not clearly defined, study design is missing elements, tracking measures are not clearly stated. | Written submission is detailed and complete. PDSA cycle includes all supplemental materials needed. All roles are identified.  All the details of study design are included. Tracking measures are clearly stated. Clear future plans for increasing the scope of the project. |
| **Final Written Proposal**  **Due: [ ]** | Assignment was late or missing. | Some elements are missing from submission, or did not include facilitator feedback. Contains grammatical errors. | Written submission is detailed and complete. Incorporated facilitator feedback. Included thoughtful and well-written introduction and conclusion. |
| **Platform Presentation**  **Due: [ ]** | Assignment was late or missing. Presentation was inaccurate or incomplete. | Presentation was mostly clear, but there were errors or confusion in presenting the problem, supporting literature, or PDSA cycle. | Identified problem was clearly explained. Evidence to support the problem and the intervention were reviewed. PDSA cycles are well thought out and explained. Interpretation and conclusions from the project were thoughtful. |
| **Peer Review of Presentations**  **Due: [ ]** |  |  | This is not a graded assignment, but must be completed by the due date. |
| **Peer Review of Group Member Participation**  **Due: [ ]** |  |  | This is not a graded assignment, but must be completed by the due date. |
