# Supplementary material for: Quality Improvement Amid a Global Pandemic: A Virtual Curriculum for Medical Students in the Time of COVID-19
Source: MedEdPORTAL. 2021 Feb 5;17:11090. doi: 10.15766/mep_2374-8265.11090 (PMC7880258; doi:10.15766/mep_2374-8265.11090)
Supplement: Supplementary file 1 — Assessment Rubrics.docxPreceptor Facilitation Instructions.docxCurricular Outline.docxPostcourse Survey Questions.docxCourse Syllabus.docxLecture Introduction to Quality Improvement.pptxLecture Introduction to Incident Command Systems.pptxStakeholder Analysis Template.xlsxProject Charter.pptxPlan-Do-Study-Act Worksheet.pdfPeer Feedback Rubric.docxPeer Review of Presentations.docx [file mep_2374-8265.11090-s001.zip › B. Preceptor Facilitation Instructions.docx]

**Facilitation Notes**

I. **Introduction**

This 4-week curriculum will serve as an introduction to quality improvement work and will be taught by distance learning. Learners will be given on introductions to Incident Command Systems and quality improvement (QI) through basic lectures and readings and will be asked to complete their own medical innovation or quality improvement intervention over the course of the 4 weeks. Learners will work in groups to research a problem or question that is clinically relevant during the current global pandemic and amenable to QI. Each week of the course will focus on different skills, including problem identification, literature review, and designing a Plan-Do-Study-Act (PDSA) cycle, and the course will culminate in a final paper and presentation of their intervention. As group facilitators, your primary role will be to provide background on QI and PDSA cycles, guide their choice of project, and provide feedback on assignments as they progress through the project. All meetings will be conducted virtually.

This course will meet the standards to fulfill an elective requirement for medical school graduation.

Facilitators chosen for this course will ideally have experience in Quality Improvement or Incident Command Systems. In the event that facilitators wish to improve their Quality Improvement knowledge prior to the administration of this course, we suggest completing the Institute for Health Care Improvement Open School Basic Certificate in Quality and Safety.^1^ There are a number of excellent quality improvement courses available through various professional organizations which could provide proficient knowledge to facilitate the course. The Certificate in Patient Safety Program offered by the Johns Hopkins Armstrong Institute^2^ and the Advanced Training Program in Clinical Quality Improvement from Intermountain Healthcare^3^ are both comprehensive and can be completed online. Additionally, we suggest pairing facilitators with less QI knowledge with more experienced facilitators so they can gain experience through repeated iterations of the curriculum.

A. **Logistics:**

**1. Technology needs:**

All students will need access to the internet, the hospital e-library system, and to the virtual classroom software used by the institution. The students have access to this on their own computers.

B. **Curriculum Administration:**
1. **Prior to Activity (Early in Week 1):**

**Background Knowledge:** Students will receive lectures on QI and Incident Command Systems and complete the recommended Institute for Healthcare Improvement (IHI) QI and patient safety modules, as well as the recommended readings.

**Group Assignments:** Groups will be pre-assigned by the instructor with the goal of having diversity in gender, race, and scholastic performance.

**2. During Activity:**

The activity will take place over 4 weeks with biweekly meetings (virtual) with the group facilitator as well as assignments due at the completion of weeks 1 through 3 and a final project and presentation due at the conclusion of week 4. As the students need real-time feedback on their assignments to proceed in weeks 1 to 3, it will be important for the group facilitator to review the assignments, provide feedback, and have meetings with each group. We expect each group facilitator to have a virtual meeting with their student group during the first two days each week to provide guidance and answer questions about course content and deliverables. Group facilitators will then meet with students again at the conclusion of the week to provide detailed feedback on the deliverables. It is anticipated that each meeting with the student groups will take 45-60 minutes. The week 4 assignment will be presented to the class in a mini-research symposium where students are able to give feedback on each other's projects.

**3. After Activity:**

The course directors will complete a rubric regarding the assignments. Each student will also complete an anonymous rubric on peer participation for their own group members.

________________________________________________________________________________

**References**:

1. Institute for Healthcare Improvement. IHI Open School Online Courses. http://www.ihi.org/OnlineCourses. Accessed September 16, 2020.
2. Armstrong Institute for Patient Safety and Quality. Online Patient Safety Certificate Program. https://www.hopkinsmedicine.org/armstrong_institute/training_services/eLearning/armstrong_institute_catalog/patient_safety_certificate.html. Accessed September 16, 2020.
3. Intermountain Healthcare: Healthcare Delivery Institute. Quality Improvement Training. https://intermountainhealthcare.org/about/transforming-healthcare/hdi/atp/. Accessed September 16, 2020.
