# Supplementary material for: Quality Improvement Amid a Global Pandemic: A Virtual Curriculum for Medical Students in the Time of COVID-19
Source: MedEdPORTAL. 2021 Feb 5;17:11090. doi: 10.15766/mep_2374-8265.11090 (PMC7880258; doi:10.15766/mep_2374-8265.11090)
Supplement: Supplementary file 1 — Assessment Rubrics.docxPreceptor Facilitation Instructions.docxCurricular Outline.docxPostcourse Survey Questions.docxCourse Syllabus.docxLecture Introduction to Quality Improvement.pptxLecture Introduction to Incident Command Systems.pptxStakeholder Analysis Template.xlsxProject Charter.pptxPlan-Do-Study-Act Worksheet.pdfPeer Feedback Rubric.docxPeer Review of Presentations.docx [file mep_2374-8265.11090-s001.zip › C. Curricular Outline.docx]

**Curricular Outline**

Week 1:

- Lectures
  - Introduction to Quality Improvement
  - Introduction to Incident Command Systems
- Assignments
  - Quality Improvement Knowledge Application Tool—Revised (QIKAT-R)
  - Brief problem identification synopsis
  - Stakeholder analysis
  - Project charter
- Suggested Readings
  - Silver article (Required)
  - Institute for Healthcare Improvement (IHI) modules
- Required Meetings
  - Facilitator feedback
  - *[insert your institution’s incident command or patient safety meetings here]*

Week 2:

- Assignments
  - Literature review
  - Process map
- Suggested Readings
  - Any suggested resources listed in Syllabus
- Required Meetings
  - Facilitator feedback
  - *[insert your institution’s incident command or patient safety meetings here]*

Week 3:

- Assignments
  - Plan-Do-Study-Act (PDSA) cycle design
  - PDSA worksheet
  - Report on PDSA implementation
- Suggested Readings
  - Any suggested resources listed in Syllabus
- Required Meetings
  - Facilitator feedback
  - *[insert your institution’s incident command or patient safety meetings here]*

Week 4:

- Assignments
  - Final written proposal
  - Platform presentation
  - Peer review of presentations
  - Peer review of own group member participation
- Suggested Readings
  - Any suggested resources listed in Syllabus
- Required Meetings
  - Facilitator feedback
  - *[insert your institution’s incident command or patient safety meetings here]*
