# Supplementary material for: Quality Improvement Amid a Global Pandemic: A Virtual Curriculum for Medical Students in the Time of COVID-19
Source: MedEdPORTAL. 2021 Feb 5;17:11090. doi: 10.15766/mep_2374-8265.11090 (PMC7880258; doi:10.15766/mep_2374-8265.11090)
Supplement: Supplementary file 1 — Assessment Rubrics.docxPreceptor Facilitation Instructions.docxCurricular Outline.docxPostcourse Survey Questions.docxCourse Syllabus.docxLecture Introduction to Quality Improvement.pptxLecture Introduction to Incident Command Systems.pptxStakeholder Analysis Template.xlsxProject Charter.pptxPlan-Do-Study-Act Worksheet.pdfPeer Feedback Rubric.docxPeer Review of Presentations.docx [file mep_2374-8265.11090-s001.zip › D. Postcourse Survey Questions.docx]

Pandemic Quality Improvement and Disaster Medicine Elective

Post-Course Survey

1. I completed all required modules, readings, lectures and project assignments.
   a. Yes
   b. No
2. This was my first experience with quality improvement work.
   a. Yes
   b. No
3. The IHI modules and readings were valuable in improving my knowledge of quality improvement.
   a. 1 (Strongly Disagree)
   b. 2 (Disagree)
   c. 3 (Neutral)
   d. 4 (Agree)
   e. 5 (Strongly Agree)
4. I found the daily hospital wide safety huddle to be a valuable learning experience.
   a. 1 (Strongly Disagree)
   b. 2 (Disagree)
   c. 3 (Neutral)
   d. 4 (Agree)
   e. 5 (Strongly Agree)
5. The Introduction to Quality Improvement lecture improved my QI knowledge.
   a. 1 (Strongly Disagree)
   b. 2 (Disagree)
   c. 3 (Neutral)
   d. 4 (Agree)
   e. 5 (Strongly Agree)
6. The Introduction to Disaster Medicine lecture improved my knowledge of disaster medicine.
   a. 1 (Strongly Disagree)
   b. 2 (Disagree)
   c. 3 (Neutral)
   d. 4 (Agree)
   e. 5 (Strongly Agree)
7. My knowledge of quality improvement has improved substantially as a result of participating in this elective.
   a. 1 (Strongly Disagree)
   b. 2 (Disagree)
   c. 3 (Neutral)
   d. 4 (Agree)
   e. 5 (Strongly Agree)
8. I found the virtual learning experience, including the software used, to be an effective way to deliver the course content.
   a. 1 (Strongly Disagree)
   b. 2 (Disagree)
   c. 3 (Neutral)
   d. 4 (Agree)
   e. 5 (Strongly Agree)
9. I felt more connected to the hospital wide efforts to combat the Covid-19 pandemic by participating in this elective.
   a. 1 (Strongly Disagree)
   b. 2 (Disagree)
   c. 3 (Neutral)
   d. 4 (Agree)
   e. 5 (Strongly Agree)
10. I would recommend this elective to my colleagues as a valuable learning experience.
    a. 1 (Strongly Disagree)
    b. 2 (Disagree)
    c. 3 (Neutral)
    d. 4 (Agree)
    e. 5 (Strongly Agree)
11. As a result of participating in this elective, I am more likely to engage in quality improvement work in the future.
    a. 1 (Strongly Disagree)
    b. 2 (Disagree)
    c. 3 (Neutral)
    d. 4 (Agree)
    e. 5 (Strongly Agree)
12. This a brand-new experience for us. Please leave comments and tell us what you thought about the elective! We are very interested in what you enjoyed and any suggestions for future improvement!
    a. Comments:
