# Supplementary material for: Quality Improvement Amid a Global Pandemic: A Virtual Curriculum for Medical Students in the Time of COVID-19
Source: MedEdPORTAL. 2021 Feb 5;17:11090. doi: 10.15766/mep_2374-8265.11090 (PMC7880258; doi:10.15766/mep_2374-8265.11090)
Supplement: Supplementary file 1 — Assessment Rubrics.docxPreceptor Facilitation Instructions.docxCurricular Outline.docxPostcourse Survey Questions.docxCourse Syllabus.docxLecture Introduction to Quality Improvement.pptxLecture Introduction to Incident Command Systems.pptxStakeholder Analysis Template.xlsxProject Charter.pptxPlan-Do-Study-Act Worksheet.pdfPeer Feedback Rubric.docxPeer Review of Presentations.docx [file mep_2374-8265.11090-s001.zip › E. Course Syllabus.docx]

**Course Syllabus: Pandemic Medicine Quality Improvement Curriculum**

**Course Directors: [ ]**

**Group Facilitators: [ ]**

**1. Introduction**

In this course you will receive an introduction to disaster medicine and quality improvement, followed by an opportunity to identify a real problem in the current global pandemic and then design a quality improvement project over four weeks. You will work in small teams to identify a current problem, perform a literature review, and design a medical innovation or intervention that you think would help the problem. You will then design a Plan-Do-Study-Act (PDSA) Quality Improvement cycle for how you would test your innovation or intervention. The culmination of your project will be a paper and a presentation of your results to your peers in a research symposium where you will have the opportunity to discuss and critique each other’s work.

**2. Learning Outcomes**

By the end of this elective, learners will be able to:

1. Apply new quality improvement and disaster medicine knowledge to a pandemic-related problem
2. Identify a small but impactful problem in the hospital or medical school related to the current public health crisis amenable to quality improvement research
3. Develop a process map and stakeholder analysis
4. Write a thorough literature review and use evidence to support an intervention or innovation
5. Using knowledge gained in the course, develop a complete project plan for a Plan-Do-Study-Act (PDSA) cycle
6. Engage with teammates to produce a final project plan and virtual platform presentation of the QI proposal

**3. Required Lectures, Readings and Materials**

**Introduction to QI:** Date and Time TBD

**Introduction to Disaster Medicine:** Date and Time TBD

**QI Article:** Silver, S. A., Harel, Z., McQuillan, R., Weizman, A. V., Thomas, A., Chertow, G. M., Nesrallah, G., Bell, C. M., & Chan, C. T. (2016). How to Begin a Quality Improvement Project. *Clinical journal of the American Society of Nephrology : CJASN*, *11*(5), 893–900. <https://doi.org/10.2215/CJN.11491015>

**4. Suggested Supplemental Materials**

**Institute for Health Care Improvement Modules^[1]^ (Quality Improvement 101-105 and Patient Safety 101-104):** <http://www.ihi.org/education/IHIOpenSchool/Courses/Pages/default.aspx>

These modules provide a basic introduction to participating in quality improvement, as well as how to design a PDSA cycle and interpret quality improvement data.

*Instructions for accessing the modules:*

*Step 1. Register for IHI*

*A. Go to* [*http://www.ihi.org/education/ihiopenschool/Pages/default.aspx*](http://www.ihi.org/education/ihiopenschool/Pages/default.aspx)

*B. In upper banner, click on “Log In/Register”*

*C. Select the “Register Now” button in the center of the page*

*D. Complete the required fields, accept the Terms of Use, and click on “Complete Registration”*

*Step 2. Access Modules*

*A. Go to* [*http://www.ihi.org/education/ihiopenschool/Pages/default.aspx*](http://www.ihi.org/education/ihiopenschool/Pages/default.aspx)

*B. On the left-hand side, select “OPEN SCHOOL: The IHI Open School is transforming health care education around the world >>”*

*C. Under “Quick Links” on the left-hand tab, select “Take a Course”*

*D. Select the course you wish to take and begin*

**Additional Resources:**

If you are interested in learning more about any of these topics you can utilize the following resources:

Institute for Healthcare Improvement (IHI) is an excellent source of learning more about PSQI Try the “Resources” tab <http://www.ihi.org/resources/Pages/default.aspx>, or take additional “Courses.”

The Agency for Healthcare Research and Quality: (AHRQ) https://www.ahrq.gov/

The Joint Commission (Accreditation body for US hospitals): https://www.jointcommission.org/standards_information/npsgs.aspx

The World Health Organization: http://www.who.int/patientsafety/en/

**5. Course Description:**

After completing the required lectures and readings, you will embark on your own project with a group of other students. During this course, you will first identify a problem amenable to quality improvement and conduct a literature review/background analysis on the question. Then you will work with your group facilitator to design an intervention, and then a basic PDSA cycle to address the problem. Your team will write up your proposal and prepare a virtual presentation to your peers. You will likely find the suggested IHI modules to be particularly useful for this task.

A. **Directions and Logistics:**

**Virtual Meetings:**

[ *INSERT YOUR HOSPITAL SYSTEM’S SARS-CoV-2 RELATED PATIENT SAFETY OR INCIDENT COMMAND MEETINGS* ]

Group facilitators may help you find additional meetings depending on your project topic.

Each group is expected to have a virtual meeting with their facilitator during the first two days of each week to receive guidance on assignments and discuss any questions about the course content. Groups will then meet with facilitators again at the conclusion of the week to receive detailed feedback on assignments. It is anticipated that each meeting will take 45-60 minutes.

**Week 1:** You will be assigned groups of 3-4 students. After completing the IHI modules, readings, and other virtual lectures, brainstorm together to identify a problem within the current pandemic crisis that you think is amenable to medical intervention or innovation and a quality improvement project. The problem should be specific, measurable, and stretchable and you will answer the first two questions from the “Model for Improvement” (Silver, 2016). You will submit a 1-2 page double-spaced synopsis of your identified problem to your facilitator by the end of the week. You should also develop a process charter (explained in the Introduction to QI lecture). Your facilitator will provide written or verbal feedback on your problem prior to the start of week 2. Your process charter is a living document for YOU; it will not be graded. It is designed to help you with this process.

**Week 2:** Perform a detailed literature review, gather information (interview relevant people, gather current protocols in place), and prepare an in-depth analysis of the problem and your planned innovation or intervention to submit to your instructor. Your literature review should include a basic process map. The group facilitator will provide written or verbal feedback on your literature review and planned intervention before moving on to week 3.

**Week 3:** Design a 1-week PDSA cycle that can be enacted to intervene on the problem. The PDSA cycle should be detailed and include all the necessary personnel, any materials you need (eg, signs, materials for teaching, forms) and details about who is going to do the data collection and the roles of all personnel. Submit a written plan for your PDSA cycle to your group facilitator by the end of the week.

**Week 4:** The final week of the project will be spent writing up your final proposal and preparing a platform presentation for your peers. Your final proposal will be submitted for grading. Your platform presentation will be uploaded for viewing by your group facilitator and peers. You will be expected to evaluate and submit feedback on other groups' presentations.

**B. Assignments:**

There will be several assignments submitted according to the timeline below. Grading criteria are outlined in each rubric. All written assignments should be submitted as Microsoft Word documents, double-spaced in Times New Roman 12-point font.

1. **Brief problem synopsis:** A synopsis of your QI question/problem will need to be submitted by the end of week 1. Your problem synopsis should be 1-2 pages.

- Identify a specific, measurable, and stretchable problem (Silver, 2016).
  - Describe the problem. Why is it a problem? What goes into the problem? Try to be as detailed as possible in describing how it is specific, measurable, and stretchable.
- Answer the first two questions from “Model for Improvement” (Silver, 2016)
  - What are we trying to accomplish (Aim)?
  - How will we know a change is an improvement (Measures)?
- Identify all the stakeholders in your synopsis.
  - Include stakeholder analysis.
- Include a process charter with your synopsis. This is not graded; it is a working document for you.

2. **Literature Review:** A complete literature review of your identified problem and planned intervention will need to be submitted by the end of week 2. Your literature review should be 5 pages, cited in APA format, and include the following sections:

- Literature review of the problem you identified (2 pages, at least 5 references)
  - You do not need to readdress the questions you answered in your problem synopsis
  - The goal of this part of the assignment is to use evidence to support your choice of problem
- Description of your planned intervention or innovation. You should describe it in detail and use evidence to support your idea (2 pages, at least 5 references). Include logistics of how it will be implemented, be sure to discuss any personnel, equipment, etc.
- A process map to help explain the contributing factors to the problem and intervention (1 page). The Introduction to QI lecture will explain how to develop a process map.

3. **PDSA Cycle Plan:** Submit a 3-4 page proposal of how you would design and implement your PDSA cycle to study your innovation or intervention. Your PDSA cycle should be submitted by the end of week 3. A PDSA worksheet is available to guide you, but does not need to be submitted.

- Identify the following roles: Team Lead, Technical Experts, Clinical/System Leader, Improvement Advisor, Executive Sponsor (Silver, 2016).
- Include all the details of your study design, including who, what, where, and how it will be implemented.
- Discuss how you will establish and track measures of improvement during the “Plan” and “Do” phase of PDSA.
- Discuss how you will learn from data during the “study” phase of PDSA.
- Explain how you plan to increase the scope of subsequent test cycles based on what you’re learning during the “act” phase of PDSA.

4. **Platform Presentation:** You and your team will need to prepare a 20-minute platform presentation that you will upload for your peers to view. Your presentation should include all the details of your problem synopsis, literature review, and PDSA cycle plan. Please do not go over the 20-minute recommended length. You may use any presentation modality that you think works best for your group, provided that it can be uploaded for other groups to view. You will need to upload this by 8am on the final day of the course, so other students can view your presentation.
5. **Final Proposal:** Using feedback from your group facilitator, make any necessary changes to your three written assignments and combine them into a final project proposal. Each section should have a distinct title (problem synopsis, literature review, PDSA cycle), and then please add an introduction and conclusion to make it a finished product.
6. **Peer Feedback on Platform Presentation:** Each individual will be expected to watch and comment on all other groups’ platform presentations (note: this is not a group assignment). Complete the rubric for each group, and submit to the course facilitator who will anonymize and distribute back to the groups.

**C. Assessment:**

Your group will receive verbal or written feedback on your problem synopsis, literature review, and PDSA cycle prior to proceeding to ensure that you are on the right track with your project. Assignments will be evaluated based on the rubric below. At the completion of the course, your group will receive the rubric completed by the course director.

Each group member will also be asked to complete an anonymous evaluation of the other group members' performances.

Final grade will be based both on the rubric (80%) and on peer assessments (20%). A final score of 65% and above is considered passing.

1. Institute for Healthcare Improvement. IHI Open School Online Courses. http://www.ihi.org/OnlineCourses. Published 2009. Updated June 10, 2019. Accessed June 10, 2020.
