# Supplementary material for: Quality Improvement Amid a Global Pandemic: A Virtual Curriculum for Medical Students in the Time of COVID-19
Source: MedEdPORTAL. 2021 Feb 5;17:11090. doi: 10.15766/mep_2374-8265.11090 (PMC7880258; doi:10.15766/mep_2374-8265.11090)
Supplement: Supplementary file 1 — Assessment Rubrics.docxPreceptor Facilitation Instructions.docxCurricular Outline.docxPostcourse Survey Questions.docxCourse Syllabus.docxLecture Introduction to Quality Improvement.pptxLecture Introduction to Incident Command Systems.pptxStakeholder Analysis Template.xlsxProject Charter.pptxPlan-Do-Study-Act Worksheet.pdfPeer Feedback Rubric.docxPeer Review of Presentations.docx [file mep_2374-8265.11090-s001.zip › K. Peer Feedback Rubric.docx]

**Peer Feedback Rubric**

Please complete the rubric below for EACH member of your group. Your responses will be anonymized and scores will be aggregated. Select comments may be edited and appear in your peers’ final course evaluations.

Group Member’s Name:

|  | **1pt** | **5pts** | **10pts** |
| --- | --- | --- | --- |
| **Workload** | Completed substantially less work than other teammates. | Did almost an equal share of the work, sometimes doing less than other group members. | Did an equal share of the work. Spearheaded certain aspects of the project. |
| **Participation in Team Meetings** | Not present at team meetings. | Present, generally on-time, occasionally offered ideas or suggestions to the group. | Present, on-time and engaged for all team meetings. Offered ideas and suggestions frequently to the group. |
| **Professionalism and Feedback** | Negative interactions with peers, did not provide respectful feedback, and/or did not accept feedback. | Mostly positive interactions with peers. Occasional difficulty providing respectful feedback or accepting feedback from peers. | Communicated clearly. Positive interactions with all peers. Provided respectful feedback and accepted feedback graciously. |

Comments: Please provide 2-3 constructive comments for your peer.
