# Supplementary material for: Quality Improvement Amid a Global Pandemic: A Virtual Curriculum for Medical Students in the Time of COVID-19
Source: MedEdPORTAL. 2021 Feb 5;17:11090. doi: 10.15766/mep_2374-8265.11090 (PMC7880258; doi:10.15766/mep_2374-8265.11090)
Supplement: Supplementary file 1 — Assessment Rubrics.docxPreceptor Facilitation Instructions.docxCurricular Outline.docxPostcourse Survey Questions.docxCourse Syllabus.docxLecture Introduction to Quality Improvement.pptxLecture Introduction to Incident Command Systems.pptxStakeholder Analysis Template.xlsxProject Charter.pptxPlan-Do-Study-Act Worksheet.pdfPeer Feedback Rubric.docxPeer Review of Presentations.docx [file mep_2374-8265.11090-s001.zip › L. Peer Review of Presentations.docx]

**Peer Review Rubric - Final Presentations**

Please complete the rubric for EACH of the other groups’ presentations.

Your Name:

Title of Project Reviewing:

| **Problem Identification** | Problem was not clearly identified or there was no explanation for why it was chosen. | Problem was mostly clear. Some confusion or lack of information as to why the problem was chosen. | Problem is clearly stated, with an explanation of why it was chosen for a QI project. |
| --- | --- | --- | --- |
| **Literature Review** | Literature review for either problem or intervention was missing. | Elements of literature review were incomplete or not clear. Some small gaps in how research supports the planned intervention. | Literature review was extensive and succinctly presented. Team made it clear how research supports their problem and planned intervention. |
| **PDSA Cycle** | >2 key elements missing. | Most of the PDSA cycle was presented, but one or two key elements were missing. | All details of PDSA cycle were presented. Roles were clearly identified. Tracking measures were identified and explained. Future plans were conveyed. |
| **Overall Presentation** | Presentation was confusing or missing large elements. | Presentation was overall clear, but some aspects of the project were not clearly presented. | Presentation was well organized and presented without errors. I had no difficulty understanding the project. |

Please include 2-3 constructive comments for the group:
